# Supplementary material for: Insights into soil nematode diversity and bacterial community of Thai jasmine rice rhizosphere from different paddy fields in Thailand
Source: PeerJ. 2024 Apr 23;12:e17289. doi: 10.7717/peerj.17289 (PMC11048080; doi:10.7717/peerj.17289)
Supplement: Supplemental Information 1 — DGO = dorsal pharyngeal gland opening; * Mean ± SD (n = 25) ** second-stage juveniles of Meloidogyne graminicola [file peerj-12-17289-s001.docx]

**Supplementary Table 1** Morphometrics of adult females of plant-parasitic nematodes obtained from roots and rhizosphere soils of *Oryza sativa* L. cv. Khao Dawk Mali 105

| Morphometric parameter | Length (µm) * | | | | |
| --- | --- | --- | --- | --- | --- |
|  | *M. graminicola*** | *Hirschmanniella* | *Pratylenchus* | *Helicotylenchus* | *Tylenchorhynchus* |
| Overall body length | 506.60 ± 62.40  (450.90-609.40) | 2,034.20 ± 183.40  (1625.37-2293.41) | 536.19 ± 61.85  (409.60-636.90) | 730.98 ± 51.32  (640.10-824.10) | 709.34 ± 49.13  (640.00-807.40) |
| Maximum body diameter | 15.80 ± 2.08  (12.20-18.70) | 32.40 ± 3.21  (28.40 - 37.80) | 24.04 ± 3.60  (18.00-29.40) | 31.10 ± 1.17  (29.10-32.90) | 22.34 ± 1.53  (19.10-24.40) |
| Tail length | 79.50 ± 7.53  (67.90-91.70) | 86.16 ± 3.88  (79.80-94.30) | 35.25 ± 4.58  (27.90-43.00) | 16.82 ± 2.73  (12.30-22.70) | 53.74 ± 6.39  (41.50-62.80) |
| (%) V position | - | 54.80 ± 3.32  (48.20-59.30) | 79.51 ± 3.14  (73.00-84.70) | 62.74 ± 0.91  (61.10-64.30) | 53.91±1.34  (51.30-55.80) |
| Hyaline tail part | 18.20 ± 1.73  (15.60-21.00) | - | - | - | - |
| Stylet length | 11.60 ± 1.71  (9.70-13.20) | 26.44 ± 2.29  (23.00-29.40) | 154.196±19.50  (123.10-188.20) | 25.76 ± 1.83  (23.10-28.60) | 20.53 ± 1.65  (18.30-23.40) |
| DGO to stylet knobs | 2.80 ± 0.84  (2.30-3.60) | 3.15 ± 0.76  (2.10-4.60) | 2.82±0.86  (1.50-4.10) | 9.08 ± 1.40  (9.10-13.60) | 2.39 ± 0.17  (2.10-2.60) |
|  |  |  |  |  |  |

DGO = dorsal pharyngeal gland opening; * Mean ± SD (n = 25) ** second-stage juveniles of *Meloidogyne graminicola*
