# Supplementary material for: Insights into soil nematode diversity and bacterial community of Thai jasmine rice rhizosphere from different paddy fields in Thailand
Source: PeerJ. 2024 Apr 23;12:e17289. doi: 10.7717/peerj.17289 (PMC11048080; doi:10.7717/peerj.17289)
Supplement: Supplemental Information 2 — *Data shown in format of r (P-value) **Correlation is significant at the 0.05 level [file peerj-12-17289-s002.docx]

**Supplementary Table 2** Spearman’s (*r*) correlations of abiotic and biotic factors.

| Abiotic factors | Biotic factors* | | | | | | | | | | | |
| --- | --- | --- | --- | --- | --- | --- | --- | --- | --- | --- | --- | --- |
|  | *Acidobacteriae* | gamma-*proteobacteria* | alpha-*proteobacteria* | *Clostridia* | *Verrucomicrobiae* | *Bacilli* | *Bacteroidia* | *Anaerolineae* | *Polyangia* | *Actinobacteria* | OTUs | Observed species |
| pH | -0.67(0.00)** | 0.36(0.18) | -0.66(0.00)** | 0.04(0.87) | 0.15(0.58) | 0.30(0.26) | 0.58(0.02)** | 0.73(0.00)** | 0.89(0.00)** | -0.61(0.01)** | -0.33(0.21) | -0.37(0.17) |
| Electrical conductivity (ds m^-1^) | -0.30(0.26) | 0.40(0.13) | 0.17(0.53) | -0.26(0.34) | -0.64(0.01)** | -0.19(0.48) | 0.44(0.09) | 0.74(0.00)** | 0.36(0.18) | -0.48(0.07) | 0.50(0.05) | 0.42(0.11) |
| Organic matter (%) | -0.67(0.00)** | 0.05(0.84) | -0.70(0.00)** | 0.40(0.14) | 0.61(0.01)** | 0.59(0.01)** | 0.52(0.04)** | 0.37(0.17) | 0.77(0.00)** | -0.08(0.76) | 0.69(0.00)** | 0.70(0.00)** |
| Available N (%) | -0.67(0.00)** | 0.13(0.64) | -0.72(0.00)** | 0.34(0.27) | 0.62(0.01) | 0.61(0.01)** | 0.53(0.04)** | 0.35(0.19) | 0.73(0.00)** | -0.15(0.59) | 0.76(0.00)** | 0.77(0.00)** |
| Total P (mg kg^-1^) | -0.70(0.00)** | 0.26(0.33) | 0.37(0.17) | 0.33(0.22) | -0.23(0.40) | 0.46(0.08) | 0.84(0.00)** | 0.60(0.01)** | 0.25(0.35) | 0.37(0.16) | 0.08(0.76) | -0.01(0.96) |
| Total K (mg kg^-1^) | -0.30(0.27) | 0.37(0.16) | 0.18(0.50) | -0.24(0.37) | -0.65(0.00)** | -0.108(0.49) | 0.43(0.10) | 0.74(0.00)** | 0.35(0.19) | -0.46(0.08) | 0.48(0.06) | 0.40(0.14) |
| Total Ca (mg kg^-1^) | -0.66(0.00)** | 0.37(0.16) | -0.66(0.00)** | 0.02(0.94) | 0.15(0.59) | 0.30(0.27) | 0.56(0.02)** | 0.69(0.00)** | 0.84(0.00)** | -0.63(0.01)** | -0.37(0.16) | -0.41(0.12) |
| Total Mg (mg kg^-1^) | -0.47(0.07) | 0.38(0.15) | 0.16(0.55) | -0.18(0.50) | -0.52(0.04)** | -0.02(0.93) | 0.60(0.01)** | 0.80(0.00)** | 0.51(0.04)** | -0.35(0.19) | 0.34(0.21) | 0.25(0.36) |
| Total Fe (mg kg^-1^) | -0.01(0.95) | 0.01(0.96) | 0.07(0.78) | 0.41(0.12) | 0.26(0.33) | 0.26(0.34) | -0.15(0.57) | -0.58(0.02)** | -0.39(0.14) | 0.51(0.05) | -0.34(0.21) | -0.34(0.21) |
| Total Mn (mg kg^-1^) | -0.87(0.00)** | 0.33(0.22) | -0.39(0.14) | 0.39(0.14) | 0.35(0.19) | 0.60(0.01)** | 0.68(0.00)** | 0.41(0.12) | 0.58(0.02)** | 0.06(0.83) | 0.59(0.02)** | 0.62(0.01)** |
| Total Cu (mg kg^-1^) | -0.81(0.00)** | 0.43(0.10) | -0.14(0.61) | 0.13(0.62) | -0.16(0.55) | 0.39(0.14) | 0.87(0.00)** | 0.89(0.00)** | 0.73(0.00)** | -0.25(0.35) | -0.05(0.84) | -0.16(0.56) |
| Total Zn (mg kg^-1^) | -0.72(0.00)** | 0.37(0.16) | -0.23(0.40) | 0.10(0.70) | -0.05(0.84) | 0.40(0.13) | 0.84(0.00)** | 0.89(0.00)** | 0.69(0.00)** | -0.34(0.29) | -0.06(0.82) | -0.14(0.60) |

*Data shown in format of *r*(*P*-value)

**Correlation is significant at the 0.05 level

**Supplementary Table 2** (continued) Spearman’s (*r*) correlations of abiotic and biotic factors.

| Abiotic factors | Biotic factors* | | | | | | | | | | | |
| --- | --- | --- | --- | --- | --- | --- | --- | --- | --- | --- | --- | --- |
|  | Rhizosphere soils | | | | | | Within roots | | | | | |
|  | *M. graminicola* | *Hirschmanniella* spp*.* | *Pratylenchus* spp*.* | *Helicotylenchus* spp*.* | *Tylenchorhynchus* spp*.* | Free living nematode | *M. graminicola* | *Hirschmanniella* spp*.* | *Pratylenchus* spp*.* | *Helicotylenchus* spp*.* | *Tylenchorhynchus* spp*.* | Free living nematode |
| pH | 0.33(0.22) | 0.30(0.26) | -0.88(0.00)** | 0.33(0.22) | 0.26(0.33) | 0.52(0.04)** | 0.58(0.02)** | 0.09(0.74) | -0.86(0.00)** | 0.35(0.20) | 0.00(1.00) | -0.68(0.00)** |
| Electrical conductivity (ds m^-1^) | 0.62(0.01)** | 0.29(0.28) | -0.77(0.00)** | 0.77(0.00)** | 0.05(0.86) | 0.18(0.50) | 0.88(0.00)** | -0.39(0.14) | -0.80(0.00)** | 0.35(0.02) | 0.70(0.00)** | -0.52(0.04)** |
| Organic matter (%) | 0.08(0.76) | 0.32(0.24) | -0.32(0.23) | -0.11(0.68) | -0.57(0.02)** | 0.45(0.08) | -0.08(0.75) | 0.42(0.11) | -0.30(0.27) | 0.34(0.21) | -0.69(0.00)** | -0.13(0.06) |
| Available N (%) | 0.10(0.70) | 0.32(0.23) | -0.33(0.22) | -0.11(0.68) | -0.57(0.02)** | 0.50(0.05) | -0.10(0.71) | 0.47(0.07) | -0.30(0.26) | 0.34(0.20) | -0.69(0.00) | 0.12(0.64) |
| Total P (mg kg^-1^) | 0.70(0.00)** | 0.78(0.00)** | -0.11(0.67) | 0.43(0.10) | -0.79(0.00)** | 0.39(0.14) | 0.12(0.66) | 0.06(0.82) | -0.17(0.53) | 0.68(0.00)** | -0.35(0.20) | 0.37(0.16) |
| Total K (mg kg^-1^) | 0.60(0.01)** | 0.30(0.26) | -0.76(0.00)** | 0.77(0.00)** | 0.06(0.80) | 0.23(0.40) | 0.87(0.00)** | -0.40(0.13) | -0.78(0.00)** | 0.35(0.19) | 0.69(0.00)** | -0.52(0.04)** |
| Total Ca (mg kg^-1^) | 0.28(0.29) | 0.26(0.34) | -0.88(0.00)** | 0.30(0.27) | -0.25(0.36) | 0.47(0.07) | 0.59(0.02)** | 0.12(0.66) | -0.83(0.00)** | 0.31(0.25) | 0.02(0.92) | -0.68(0.00)** |
| Total Mg (mg kg^-1^) | 0.75(0.00)** | 0.53(0.03)** | -0.75(0.00)** | 0.83(0.00)** | -0.12(0.67) | 0.22(0.41) | 0.73(0.00)** | -0.36(0.18) | -0.81(0.00)** | 0.58(0.02)** | 0.46(0.08) | -0.36(0.178) |
| Total Fe (mg kg^-1^) | -0.36(0.18) | -0.11(0.68) | 0.80(0.00)** | -0.76(0.00)** | -0.37(0.16) | 0.12(0.65) | -0.68(0.00)** | 0.35(0.18) | 0.74(0.00)** | -0.35(0.20) | -0.68(0.00)** | 0.62(0.01)** |
| Total Mn (mg kg^-1^) | 0.32(0.23) | 0.45(0.08) | -0.21(0.44) | -0.09(0.74) | -0.84(0.00)** | 0.73(0.00)** | 0.00(0.99) | 0.34(0.20) | -0.24(0.38) | 0.35(0.20) | -0.67(0.00)** | 0.00(0.97) |
| Total Cu (mg kg^-1^) | 0.75(0.00)** | 0.65(0.00)** | -0.76(0.00)** | 0.66(0.00)** | -0.52(0.04)** | 0.53(0.03)** | 0.56(0.02)** | -0.15(0.58) | -0.81(0.00)** | 0.70(0.00)** | 0.00(0.97) | -0.31(0.26) |
| Total Zn (mg kg^-1^) | 0.69(0.00)** | 0.58(0.02)** | -0.78(0.00)** | 0.65(0.00)** | 0.04(0.07) | 0.49(0.06) | 0.54(0.03)**00 | -0.04(0.88) | -0.81(0.00)** | 0.69(0.00)** | 0.00(0.98) | 0.34(0.20) |

*Data shown in format of *r*(*P*-value)

**Correlation is significant at the 0.05 level
