# Supplementary material for: Insights into soil nematode diversity and bacterial community of Thai jasmine rice rhizosphere from different paddy fields in Thailand
Source: PeerJ. 2024 Apr 23;12:e17289. doi: 10.7717/peerj.17289 (PMC11048080; doi:10.7717/peerj.17289)
Supplement: Supplemental Information 3 — *Data shown in format of r (P-value) **Correlation is significant at the 0.05 level *** Correlation is significant at the 0.01 level [file peerj-12-17289-s003.docx]

**Supplementary Table 3** Spearman’s (*r*) correlations of top 10 bacterial class and nematodes in soils and roots .

| Bacterial class | Nematodes * | | | | | | | | | | | |
| --- | --- | --- | --- | --- | --- | --- | --- | --- | --- | --- | --- | --- |
|  | Rhizosphere soils | | | | | | Within roots | | | | | |
|  | *M. graminicola* | *Hirschmanniella* spp*.* | *Pratylenchus* spp*.* | *Helicotylenchus* spp*.* | *Tylenchorhynchus* spp*.* | Free living nematode | *M. graminicola* | *Hirschmanniella* spp*.* | *Pratylenchus* spp*.* | *Helicotylenchus* spp*.* | *Tylenchorhynchus* spp*.* | Free living nematode |
| *Acidobacteriae* | -0.30(0.62) | -0.70(0.18) | 0.44(0.45) | -0.11(0.85) | 0.78(0.11) | -0.90 (0.00)*** | -0.40(0.50) | -0.3(0.62) | 0.44(0.45) | -0.35(0.55) | 0.35(0.55) | 0.05(0.93) |
| gamma-*proteobacteria* | 0.00(1.00) | 0.30(0.62) | -0.11(0.85) | -0.33(0.58) | -0.44(0.45) | 0.70(0.18) | 0.50(0.39) | 0.30(0.62) | -0.11(0.85) | -0.35(0.55) | 0.00(1.00) | -0.20(0.74) |
| alpha-*proteobacteria* | 0.50(0.39) | 0.30(0.262) | 0.44(0.45) | 0.22(0.71) | 0.11(0.85) | -0.30(0.62) | 0.00(1.00) | -0.70(0.18) | 0.44(0.45) | 0.00(1.00) | 035(0.55) | 0.56(0.32) |
| *Clostridia* | -0.10(0.87) | 0.60(0.28) | 0.44(0.45) | -0.44(0.45) | -0.89(0.04)** | 0.60(0.28) | -0.30(0.62) | 0.50(0.39) | 0.44(0.45) | 0.00(1.00) | -0.70(0.18) | 0.56(0.32) |
| *Verrucomicrobiae* | -0.70(0.18) | -0.30(0.62) | 0.11(0.85) | -0.44(0.45) | -0.22(0.71) | 0.00(1.00) | -0.60(0.28) | 0.80(0.14) | 0.11(0.85) | 0.00(1.00) | -0.70(0.18) | -0.05(0.93) |
| *Bacilli* | 0.30(0.62) | 0.80(0.10) | -0.22(0.71) | 0.22(0.71) | -0.89(0.04)** | 0.80(0.10) | -0.10(0.87) | 0.30(0.62) | -0.22(0.71) | 0.70(0.18) | -0.70(0.18) | 0.35(0.55) |
| *Bacteroidia* | 0.60(0.28) | 0.90(0.03)** | -0.44(0.45) | 0.44(0.45) | -0.78(0.11) | 0.90(0.03)** | 0.30(0.62) | 0.00(1.00) | -0.44(0.45) | 0.70(0.18) | -0.35(0.55) | 0.20(0.74) |
| *Anaerolineae* | 0.70(0.18) | 0.70(0.18) | -0.78(0.11) | 0.67(0.21) | -0.44(0.45) | 0.80(0.10) | 0.60(0.28) | -0.20(0.74) | -0.78(0.11) | 0.70(0.18) | 0.00(1.00) | -0.15(0.80) |
| *Polyangia* | -0.35(0.55) | 0.30(0.61) | -0.91(0.02)** | 0.51(0.37) | -0.22(0.71) | 0.66(0.21) | 0.56(0.32) | 0.05(0.93) | -0.91(0.02)** | 0.54(0.34) | 0.00(1.00) | -0.5(0.39) |
| *Actinobacteria* | -0.30(0.62) | 0.20(0.74) | 0.89(0.04)** | -0.44(0.45) | -0.44(0.45) | -0.20(0.74) | -0.90(0.03)** | 0.30(0.62) | 0.89(0.04)** | 0.00(1.00) | -0.70(0.18) | 0.87(0.05) |

*Data shown in format of *r*(*P*-value)

**Correlation is significant at the 0.05 level

*** Correlation is significant at the 0.01 level
