# Supplementary figures and images for: Insights into soil nematode diversity and bacterial community of Thai jasmine rice rhizosphere from different paddy fields in Thailand
Source: PeerJ. 2024 Apr 23;12:e17289. doi: 10.7717/peerj.17289 (PMC11048080; doi:10.7717/peerj.17289)

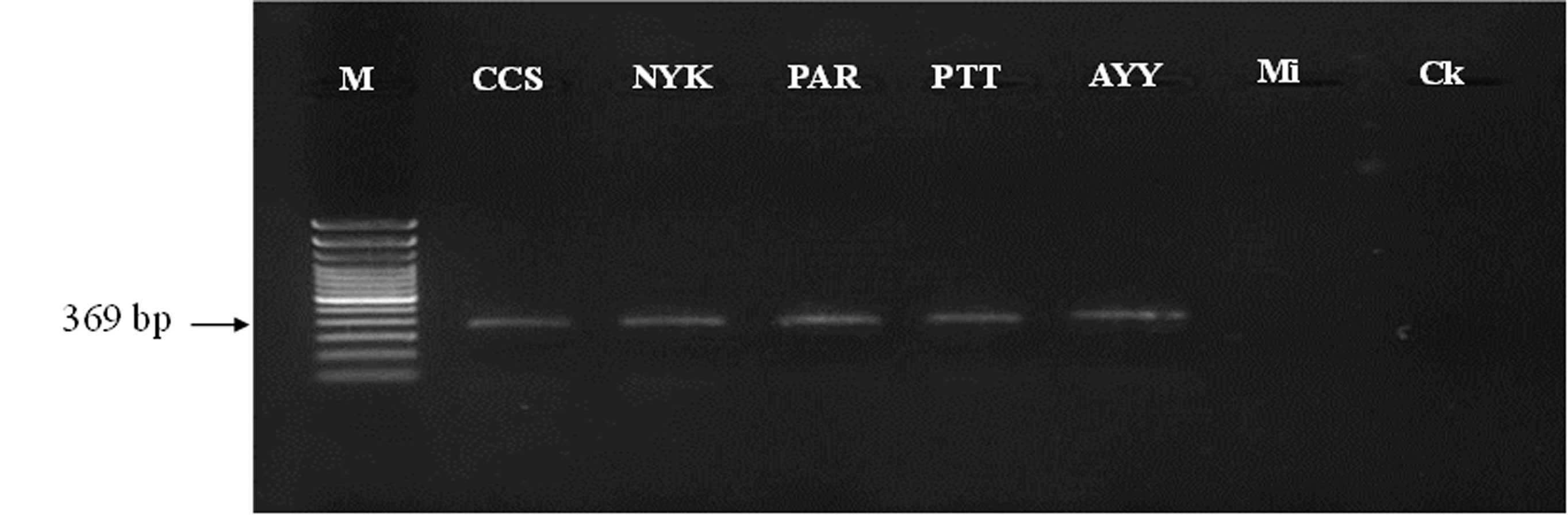

Supplement: Supplemental Information 4 — Lane 1: marker (100 bp DNA Ladder RTU); M, Lane 2: Chachoengsao; CCS, Lane 3: Nakhon Nayok; NYK , Lane 4 : Prachin Buri; PAR, Lane 5: Pathum Thani; PTT, Lane 6: Phra Nakhon Si Ayutthaya; AYY, Lane 7: M. incognita; Mi, Lane 8: negative control; Ck. [file peerj-12-17289-s004.jpg]

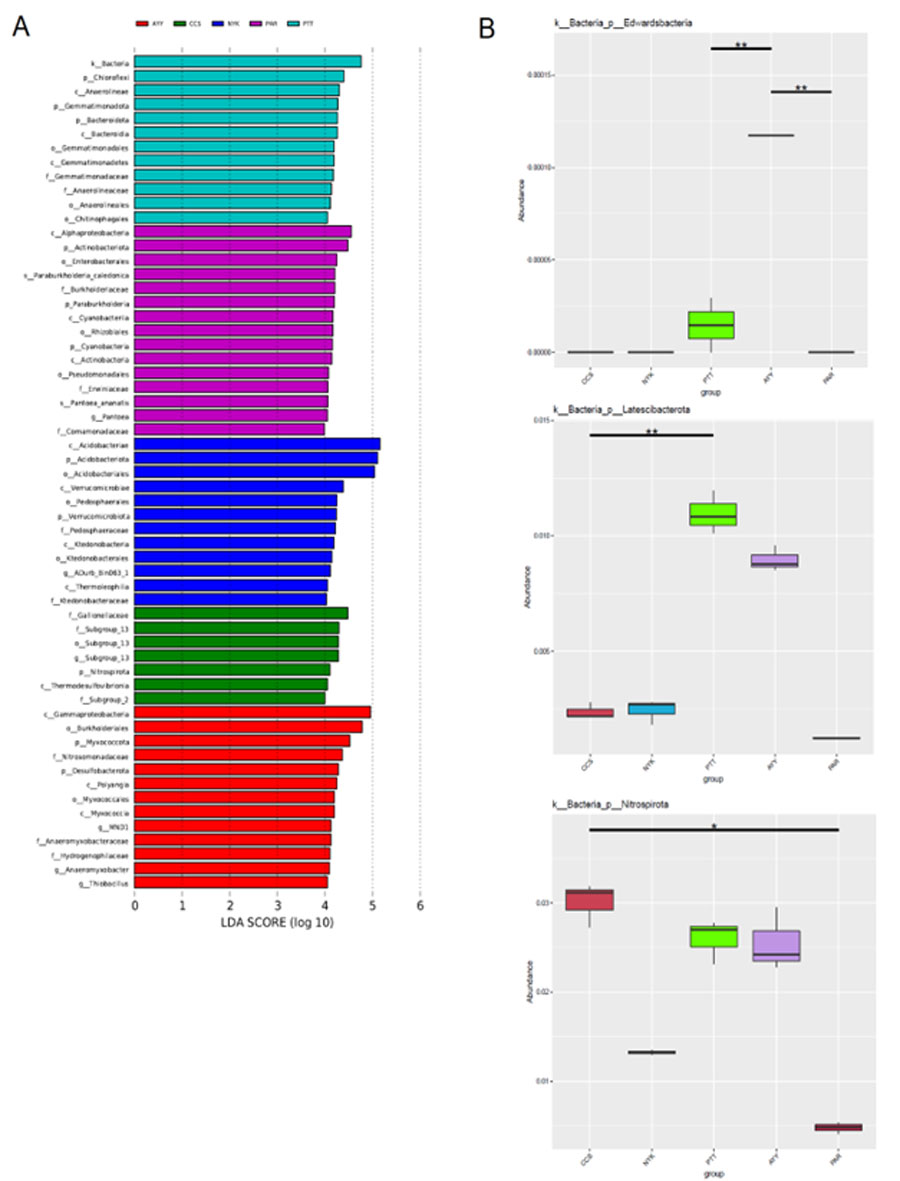

Supplement: Supplemental Information 5 — (A) Histogram of the LDA scores generated for groups with differential abundance among the bacterial communities of each site. (B) Between-group analysis, a double asterisk represents vary significant variation (p < 0.01), and a single asterisk represents significant variation (p < 0.05). [file peerj-12-17289-s005.jpg]

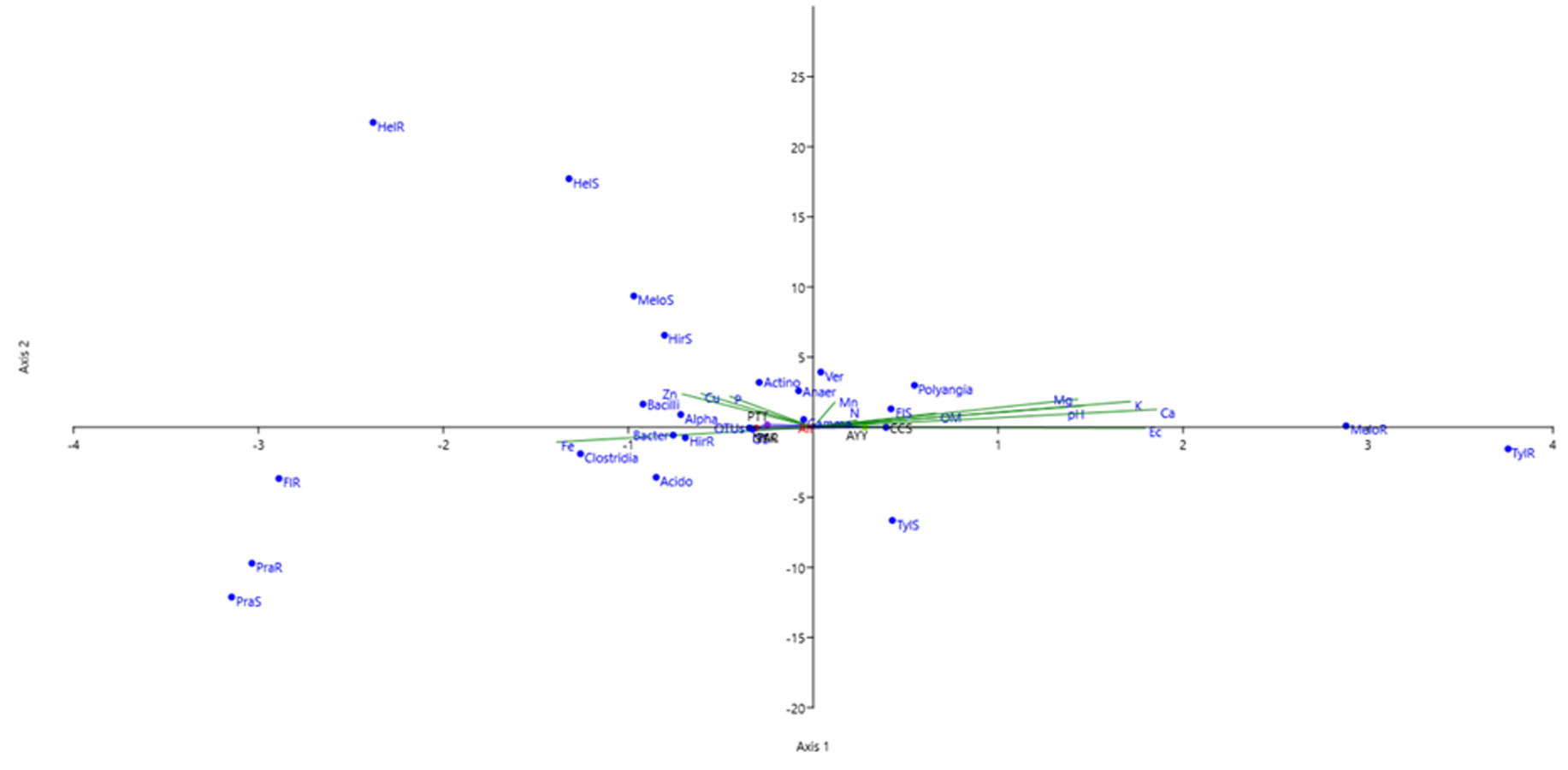

Supplement: Supplemental Information 6 — The influence of environmental factors on bacterial community structure is indicated by green line. The length of each line represents the impact of the corresponding environmental factor on the distribution of bacterial community. The longer line indicated the greater effect. OS, Observed species; Acido, Acidobacteriae; Gamma, Gamma-proteobacteria; Alpha, Alpha-proteobacteria;C; Ver, Verrucomicrobiae; Bacter, Bacteroidia; Anaer, Anaerolineae; Actino, Actinobacteria; MeloS, M. graminicola in rhizosphere soils; HirS, Hirschmanniella spp. in rhizosphere soils; PraS, Pratylenchus spp. in rhizosphere soils; HelS, Helicotylenchus spp. in rhizosphere soils; TylS, Tylenchorhynchus spp. in rhizosphere soils; FlS, Free living nematode in rhizosphere soils; MeloR, M. graminicola within roots; HirR, Hirschmanniella spp. within roots; PraR, Pratylenchus spp. within roots; HelR, Helicotylenchus spp. within roots; TylR, Tylenchorhynchus spp. within roots; FlR, Free living nematode within roots; CCS, sampling from Chachoengsao province; NYK, sampling from Nakhon Nayok province; PTT, sampling from Pathum Thani province; AYY, sampling from Phra Nakhon Si Ayutthaya province; PAR, sampling from Prachin Buri province. [file peerj-12-17289-s006.jpg]
